# The complete chloroplast genome of *Rubus setchuenensis*, an edible and medicinal dual-purpose wild plant

**DOI:** 10.1080/23802359.2021.2013740

**Published:** 2022-01-24

**Authors:** Ying-an Zhu, Chunhua Li, Shiyu Wang, Liping Lei, Jingxiu Xiao

**Affiliations:** a College of Landscape and Horticulture, Yunnan Agricultural University, Kunming, China; b Economic Crop Workstation of Lijiang City, Lijiang, China; c Research Institute of Gardening and Landscape of Kunming, Kunming, China; d College of Resources and Environment, Yunnan Agricultural University, Kunming, China

**Keywords:** Chloroplast genome sequence, Rosaceae, phylogeny

## Abstract

*Rubus setchuenensis* Bureau et Franch. is important in phylogeny and evolution amongst genus *Rubus* L. (Rosaceae) plants. The chloroplast genome of *R. setchuenensis* reported in this study is 156,231 bp in size, with an average GC content of 37.19%. The complete chloroplast genome has a typical quadripartite structure, including a large single copy (LSC) region (85,829 bp) and a small single copy (SSC) region (18,860 bp), which are separated by a pair of inverted repeats (IRs, 25,771 bp). This plastome contains 129 different genes, including 85 protein-coding genes, 36 tRNA genes, and 8 rRNA genes. The phylogenetic analysis of 20 chloroplast genomes from genera *Fragaria*, *Rosa* and *Rubus* of the family Rosaceae suggested that *R. setchuenensis* clustered into one clade with the other three species of section *Malachobatus* Focke, and then grouped with four species of section *Idaeobatus* Focke, while species from *Fragaria* and *Rosa* were classified into a group, separately.

*Rubus setchuenensis* Bureau et Franch. belongs to genus *Rubus* L., section *Malachobatus* Focke, subsection *Moluccani* (Focke) Yu et Lu in the family Rosaceae, it is widely distributed in temperate zone, subtropical and tropical zone in China, such as Hubei, Hunan, Guangxi, Sichuan, Yunnan and Guizhou province, with elevation between 500 and 3000 m (Robertson 1974; Yu and Lu 1985; Thompson 1995; Lu and Boufford 2003). Its fresh black fruits can be used for food, and the roots can be used for medicine and tannin extraction. Its bark can be used as a raw material for papermaking, and the seeds can be extracted oil. The most prominent trait is that *R. setchuenensis* is unarmed with no prickles on the stems and twigs of deciduous shrubs. Therefore, it can be used as an important germplasm resource for hybridization to breed thornless blackberry varieties. However, it is extremely difficult to delimit species of genus *Rubus* because of complex morphological variations (Alice and Campbell 1999), inter- and intraspecific hybridization, polyploidization and apomixis. Frequently, *R. setchuenensis* was confused with some others related kinship species in the section *Malachobatus* Focke, subsection *Moluccani* (Focke) Yu et Lu. The chloroplast genome data will help the development of DNA barcode for *Rubus* L., but the chloroplast genome of *R. setchuenensis* has not been published. Therefore, we reported the complete sequence of chloroplast genome of *R. setchuenensis* here to help construct the phylogeny and evolution of *Rubus* L.

The fresh leaves of *R. setchuenensis* were collected from Cuihua town, Daguan County, Zhaotong City, Yunnan Province of China (geospatial coordinates: 27°45′14″N, 103°53′51″E; altitude: 1123 m). The sheets of vouchered specimen, Zhu-20201014R03, had been stored at the herbarium HHP-YAU (Herbarium of Horticultural plants, College of Landscape and Horticulture, Yunnan Agricultural University). Total genomic DNA was extracted from fresh leaves by using DNA Plantzol Reagent (Invitrogen, Carlsbad, CA, USA) to construct chloroplast DNA libraries. The extracted DNA was sequenced by Illumina HiSeq Sequencing System (Illumina, San Diego, CA) and shotgun library was constructed. About 2.29 Gb pair-end (150 bp) raw reads were obtained and the low-quality sequences were filtered by using CLC Genomics Workbench v8.0 (CLC Bio, Aarhus, Denmark) to get high-quality clean reads. NOVOPlasty software (Dierckxsens et al. 2017) was used to align and assemble cp genome with *R. niveus* Thunb (MT576936) served as the reference. The complete chloroplast genome of *R. setchuenensis* was automatically annotated by using CpGAVAS (Liu et al. 2012) and then adjusted and confirmed with Geneious 9.1 (Kearse et al. 2012). The complete chloroplast genome had been submitted to the GenBank under the accession number of MZ352080. To ascertain the phylogenetic status of *R. setchuenensis*, the complete chloroplast genome sequences of 20 species from three genus of family Rosaceae, *Fragaria*, *Rosa* and *Rubus*, were aligned by using MAFFT (Katoh and Standley 2013), and a maximum likelihood (ML) tree was reconstructed with RAxML8 (Stamatakis 2014).

The chloroplast genome of *R. setchuenensis* is 156,231 bp in size, with an average GC content of 37.19%, exhibiting a typical quadripartite structure comprising a large single-copy (LSC) region of 85,829 bp and a small single-copy (SSC) region of 18,860 bp separated by a pair of identical inverted repeat regions (IRs) of 25,771 bp each. The chloroplast genome contains 129 genes (112 unique), including 85 protein-coding genes (79 unique), 36 tRNA genes (29 unique), and 8 rRNA genes (4 unique).

The ML phylogenetic tree showed that *R. setchuenensis* was mostly related to *R. kawakamii*, it clustered into one clade with the other three species of section *Malachobatus* Focke, and then grouped with four species of section *Idaeobatus* Focke, and six species of genera *Fragaria* and *Rosa* from Rosaceae formed a monophyletic group independently (Figure 1). This research lays the foundation for further understanding of the chloroplast genome information of *Rubus* plants, and provides important information for the development and utilization of peculiar plant resources.

**Figure 1.**
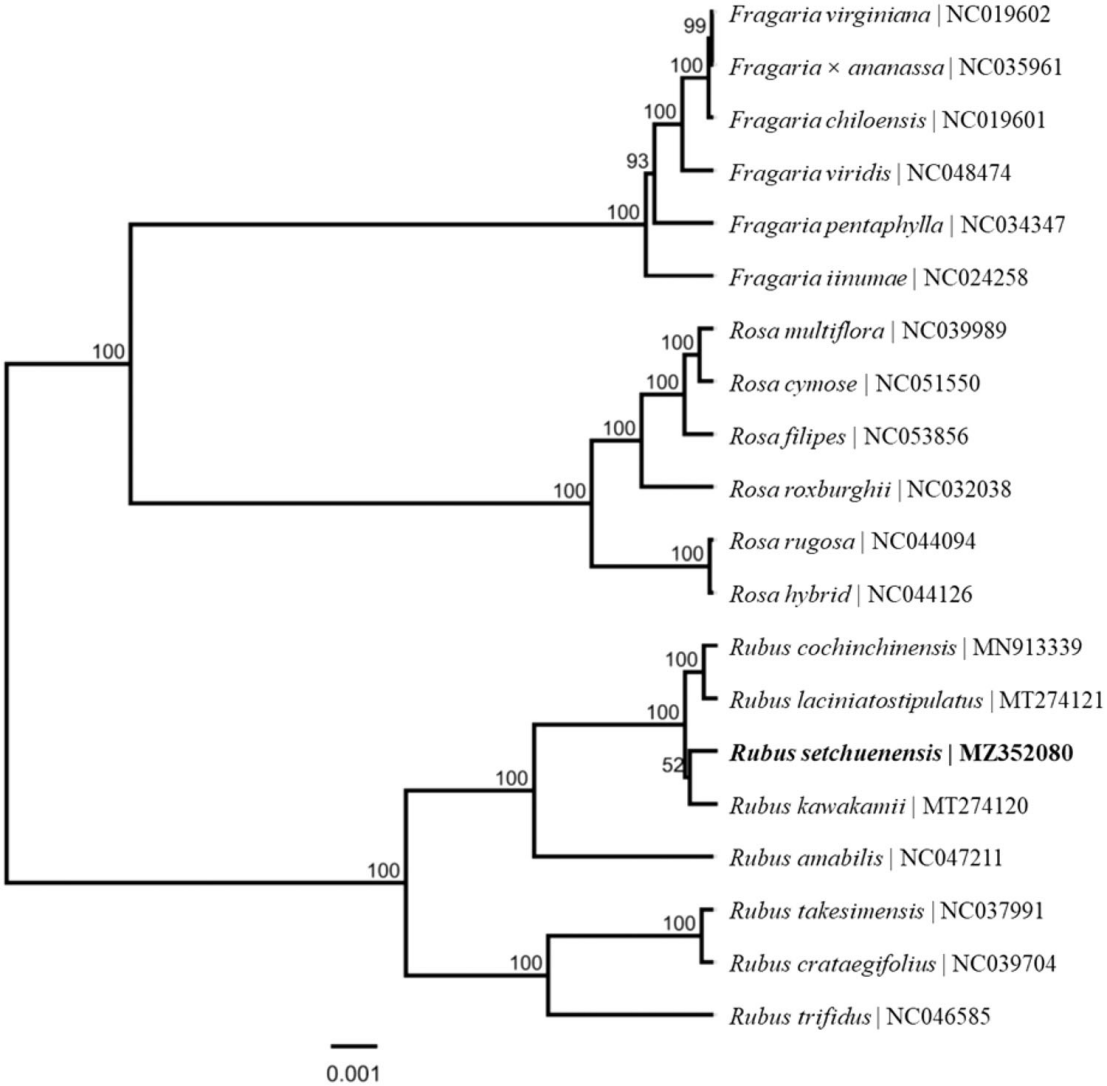
Phylogenetic relationships of 20 species from *Fragaria*, *Rosa* and *Rubus* of Rosaceae based on the complete chloroplast genome sequences. Bootstrap percentages are indicated for each branch.

## Data Availability

The complete chloroplast genome generated for this study has been deposited in GenBank with accession number MZ352080, which is openly available in GenBank of NCBI at website (https://www.ncbi.nlm.nih.gov/). All high-throughput sequencing data files are available from the GenBank Sequence Read Archive (SRA) with accession number: SRR14757449. The associated BioProject and Bio-Sample numbers are PRJNA735803 and SAMN19602743 respectively.
